# Effectiveness and safety of traditional Chinese medicine *Shaoyao Gancao Tang* for the treatment of restless leg syndrome: A protocol for systematic review and meta-analysis

**DOI:** 10.1097/MD.0000000000022401

**Published:** 2020-10-02

**Authors:** Yunhui Chen, Wei Huang, Lizhou Liu, Steve Tumilty, Dan Liu, Yanyan You, Chuan Zheng, George David Baxter

**Affiliations:** a College of Basic Medicine/Hospital of Chengdu University of Traditional Chinese Medicine, Chengdu, China; b Centre for Health, Activity, and Rehabilitation Research, School of Physiotherapy, University of Otago, Dunedin, New Zealand; c West China Hospital, Sichuan University, South Renmin Road, Wu Hou District, Chengdu, China.

**Keywords:** protocol, restlessness leg syndrome, *Shaoyao Gancao Tang*, systematic review

## Abstract

**Background:**

A growing body of clinical trials has demonstrated that traditional Chinese medicine *Shaoyao Gancao Tang* may improve restlessness leg syndrome (RLS). This review aims to systematically assess its effectiveness and safety in the treatment of patients with RLS.

**Methods:**

Eight databases will be searched from the inception to 31 August 2020, including the Chinese Biological Medicine Database, China National Knowledge Infrastructure, Wanfang Database, VIP Information Database, the Cochrane Library, PubMed, EMBASE, and the Web of Science. All published randomized controlled trials that meet the prespecified eligibility criteria will be included. The primary outcomes include the changes in the International Restless Legs Syndrome Rating Scale and the restless sensation assessed by visual analog scales, and the secondary outcomes include effective rate, adverse event rate, quality of life measures, and improvement in the sleep quality index. Study selection, data extraction, and assessment of bias risk will be conducted independently by 2 reviewers. Data synthesis will be carried out with RevMan software (V.5.3.5). Subgroup and sensitivity analysis will be performed when necessary. The strength of the evidence will be assessed by the Grading of Recommendations Assessment, Development and Evaluation System.

**Results:**

A high-quality synthesis of current evidence of *Shaoyao Gancao Tang*'s effectiveness and safety for patients with RLS will be provided.

**Conclusion:**

This systematic review will provide evidence of whether *Shaoyao Gancao Tang* is an effective and safe intervention for RLS.

## 1 Introduction

Restless leg syndrome (RLS), also known as Willis-Ekbom Disease, is a common, chronic, and neurological disease featured by an overwhelming and irresistible urge to move the legs. It often appears or aggravates at night or in resting situations, and is temporarily alleviated with movements.^[1]^ Approximately 5.0% to 14.3% of the general population suffer from RLS, females are affected twice as often as males, and the prevalence increases with age.[^2^,^3^,^4^] Studies show that RLS frequently interrupts patients’ sleep, causes insomnia, associates with hypertension, cardiovascular diseases, and cerebrovascular diseases, triggers anxiety and depression, thus impairs the quality of life.[^5^,^6^,^7^,^8^,^9^,^10^] Currently, dopamine agonist medications such as pramipexole, ropinirole, and rotigotine have been approved by the US Food and Drug Administration to treat RLS; however, their side effects, including sleepiness, dizziness, or augmentation during medication, reduce their compliance.[^11^,^12^]

*Shaoyao Gancao Tang* (also known as Shakuyakukanzoto in Japan) is a classic formulation of traditional Chinese medicine (TCM) and was first recorded in the *Treatise on Cold Damage* written by *Zhongjing Zhang* (150–219 AD) for halting spasms and cramps of legs. It comprises *Paeonia lactiflora* (*P lactiflora*) and *Glycyrrhiza uralensis* (*G uralensis*) at a ratio of 1:1. This formulation has long been used in TCM to treat a wide variety of diseases, such as myospasm, neuralgia, headache, ischialgia, spastic abdominal pain, dysmenorrhea, and cancerous pain,^[13]^ and its pharmacological effects, including spasmolysis, analgesic effect, anti-inflammatory, sedation, and neuroprotection, have been well-documented.[^14^,^15^,^16^,^17^] In recent years, an increasing number of clinical trials has been conducted and published to demonstrate the effectiveness of *Shaoyao Gancao Tang* for the treatment of RLS.[^18^,^19^,^20^,^21^,^22^,^23^]

However, no systematic review addressing the effectiveness and safety of *Shaoyao Gancao Tang* for the treatment of patients with RLS has ever been published. Herein, we propose a protocol for a systematic review to evaluate the evidence of *Shaoyao Gancao Tang* for RLS treatment and to provide necessary information for the decision making of patients, physicians, and investigators concerned.

## 2 Methods

### 2.1 Study registration

This meta-analysis has been registered on PROSPERO (www.crd.york.ac.uk /prospero/) with number CRD42020173520. It will follow the reporting guidelines and criteria set in the Preferred Reporting Items for Systematic Reviews and Meta-analyses (PRISMA) statement checklist.^[24]^ Ethical approval is unnecessary for this study as it only involves the data of previous studies.

### 2.2 Selection criteria

#### 2.2.1 Types of studies

Only randomized clinical trials (RCTs) will be included. The literature of non-RCTs, animal experiments, case reports, reviews, and conference proceedings will be excluded.

#### 2.2.2 Participants

Patients with RLS (as diagnosed using any recognized diagnostic criteria of RLS and including primary RLS and secondary RLS) will be included in the study. There will be no restrictions on the gender, nation, and duration of the disease.

#### 2.2.3 Types of interventions

RLS patients treated with *Shaoyao Gancao Tang* alone or in any combination with conventional treatments will be included. There is no restriction regarding the conventional regimen.

#### 2.2.4 Types of comparators

RLS patients treated with conventional (the same conventional regimen as intervention group in the same original study), other herbal formulations, placebo, or no treatment will be included.

#### 2.2.5 Types of outcome measures

The primary outcomes include the changes in the International Restless Legs Syndrome Rating Scale and the restless sensation assessed by visual analog scales; and the secondary outcomes include effective rate, adverse event rate, quality of life measures, and improvement in the sleep quality index.

### 2.3 Search strategy

Two reviews will search the following databases independently from the inception to August 31, 2020: the Cochrane Library, PubMed, EMBASE, Web of Science, the Chinese Biological Medicine Database, China National Knowledge Infrastructure, Wanfang Database, and VIP information database. The search terms include restlessness leg syndrome, RLS, Willis-Ekbom Disease, periodic leg movement, quiescegenic focal akathisia, Shaoyao Gancao Tang, SG Tang, Shakuyakukanzoto, SKT, Paeonia lactiflora and Glycyrrhiza uralensis decoction, and Chinese Peony and Licorice Combination. There is no limitation on the language of publication or publication period. The manual screening will also be conducted to identify the additional studies from the reference list. The search strategy for PubMed is presented in Table 1, and corresponding modification will be made upon the requirement of other databases.

**Table 1:**
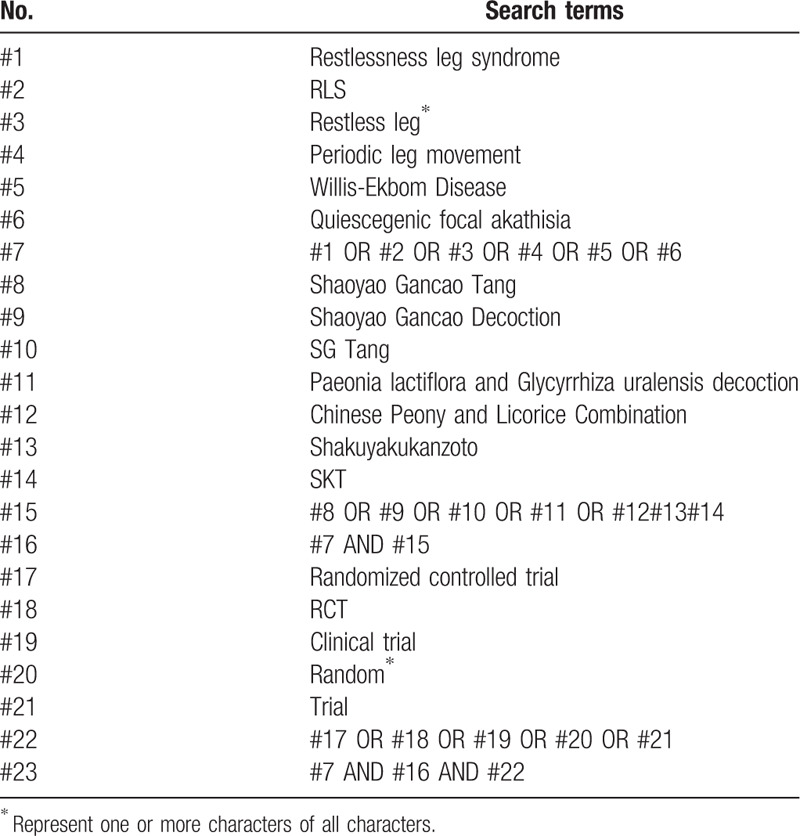
Search strategy for the PubMed.

### 2.4 Study selection and data extraction

**Figure 1:**
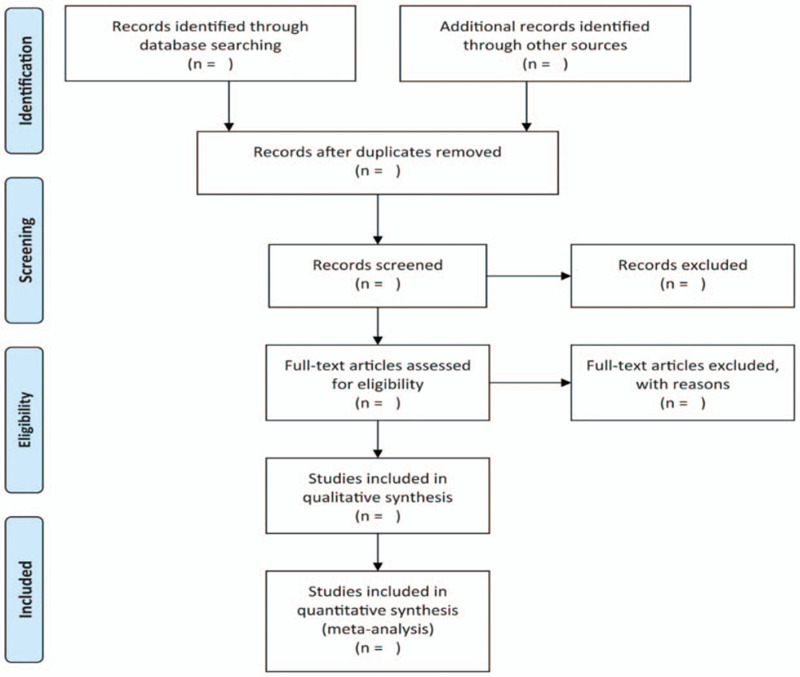
Flowchart of study selection.

Two independent reviewers will perform literature screening, study selection, and data extraction. The literature obtained will be input into EndnoteX9 for the title and abstract screening, the duplications and studies that do not meet the prespecified inclusion criteria will be excluded. The final included studies will be determined after reading the full text of the remaining studies. The corresponding author will be contacted if the full text is not available. Any disagreements will be settled by a third investigator. The process of study selection is presented in a PRISMA flow chart (Fig. 1).

The data extraction will be conducted in a standard form by 2 reviews independently, including reference ID, the name of the first author, year of publication, name of the journal, design of the study, characteristics of the patient, details of the trial intervention, details of control intervention, outcome measurements, and adverse events. Any disagreements will be settled via a discussion with a third reviewer. All data will be cross-checked and imported into RevMan software (V.5.3). If the data provided in the included study is unclear or missing, the corresponding author will be contacted by email.

### 2.5 Assessment of risk of bias

Two reviewers will independently assess the risks of publication bias for every included study using the Cochrane Handbook Version 5.2.0. in terms of random sequence generation, allocation concealment, blinding of participants and personnel, blinding of outcome assessment, incomplete outcome data, selective reporting, and other bias. Each domain will be graded as high, unclear, or low risk of bias.^[25]^ The assessment results will be cross-checked, and third-party experts will be consulted for any disagreement. A funnel plot will be conducted to assess publication bias when the included studies are more than 10.

### 2.6 Measures of treatment effect

Two reviewers will independently synthesize and statistically analyze efficacy data using RevMan 5.3. A risk ratio or odds ratio with 95% confidence intervals will be utilized for dichotomous data, while a mean difference or standard mean difference with 95% confidence intervals will be employed for continuous data. Standard mean difference will be applied if different assessment tools are used.

### 2.7 Dealing with missing data

Reviewers will contact the corresponding author by email when the required data is unclear or missing. If data is still unattainable, reviewers will exclude the study from the analysis. A sensitivity analysis will be conducted to assess the potential impact of missing data.

### 2.8 Assessment of heterogeneity

*χ* ^*2*^ test and *I* ^2^ statistic will be used for statistical heterogeneity investigation. If heterogeneity is low (*I* ^2^ < 50%), the fixed-effect model will be used; and for moderate heterogeneity (50% < *I* ^2^ < 75%), the random-effects model will be adopted. A meta-analysis will not be performed when heterogeneity is considerably high (*I* ^2^ > 75%).

### 2.9 Data synthesis

In line with the Cochrane guideline, the fixed-effects model will be utilized for the pooled data if heterogeneity is deemed low, and the random-effect model will be employed if heterogeneity is deemed moderate. Subgroup analysis or meta-regression will be performed to assess the potential sources with reasonable explanations if heterogeneity is considerably high. The statistical significance is defined as *P* < .05. If the meta-analysis is not feasible, a narrative description of the results will be provided.

### 2.10 Subgroup analysis and sensitivity analysis

If the necessary data are available, subgroup analyses will be conducted for the subtype of RLS (primary/secondary), different intervention approaches, duration, patient gender (male/female), and outcome measurements. After the quality assessment of the included literature, sensitivity analysis will be carried out if there are possible low-quality studies. We will observe the fluctuation of termination by changing the research (incorporating or excluding a study) and reanalyzing the simulated missing data.

### 2.11 Grading the quality of evidence

The strength of the evidence for primary outcome measurements will be assessed by the Grading of Recommendations Assessment, Development and Evaluation (GRADE) into high, moderate, low, or very low.[^26^,^27^]

## 3 Discussion

RLS is characterized by the irresistible urge to move the legs that frequently occurs and develops in rest or at night, interfering with rest and sleep and leading to poor quality of life and productivity. Recent years have witnessed that a growing body of clinical trials has been conducted to investigate *Shaoyao Gancao Tang*'s effect on RLS, and the results indicate that it may be an effective and safe therapeutic agent for RLS. To the best of our knowledge, this is the first systematic review to investigate the clinical effectiveness and safety of *Shaoyao Gancao Tang* for the treatment of RLS. RCTs with no limits on the language will be extracted and synthesized to provide evidence for patients, clinicians, and investigators. However, such potential limitations as gender and age of patients, and the types and duration of RLS may cause moderate to high heterogeneity, and we would give full consideration of these factors to plan and power our study appropriately.

### 3.1 Uncited references

[^4^,^10^].

## Author contributions

**Conceptualization:** Yunhui Chen, George David Baxter

**Data curation:** Wei Huang, Lizhou Liu, Dan Liu

**Formal analysis:** Yanyan You, Dan Liu, Chuan Zheng

**Funding acquisition:** Chuan Zheng

**Investigation:** Yunhui Chen, Yanyan You, Dan Liu

**Methodology:** Yunhui Chen, Lizhou Liu, Steve Tumilty, George David Baxter

**Project administration:** Yunhui Chen, Wei Huang

**Supervision:** Steve Tumilty, George David Baxter

**Validation:** Yunhui Chen, Wei Huang, Chuan Zheng

**Writing – original draft:** Yunhui Chen

**Writing – review & editing:** Yunhui Chen, Lizhou Liu, Steve Tumilty, George David Baxter
